# Association of Oral Health Conditions in Adolescents with Social Factors and Obesity

**DOI:** 10.3390/ijerph19052905

**Published:** 2022-03-02

**Authors:** Jana Schmidt, Mandy Vogel, Tanja Poulain, Wieland Kiess, Christian Hirsch, Dirk Ziebolz, Rainer Haak

**Affiliations:** 1 Department of Cariology, Endodontology and Periodontology, University of Leipzig, 04103 Leipzig, Germany; dirk.ziebolz@medizin.uni-leipzig.de (D.Z.); rainer.haak@medizin.uni-leipzig.de (R.H.); 2 LIFE Leipzig Research Center for Civilization Diseases, LIFE Child, University of Leipzig, 04103 Leipzig, Germany; tanja.poulain@medizin.uni-leipzig.de (T.P.); wieland.kiess@medizin.uni-leipzig.de (W.K.); 3 Department of Women and Child Health, Hospital for Children and Adolescents and Center for Pediatric Research (CPL), University of Leipzig, 04103 Leipzig, Germany; 4 Department of Pediatric and Preventive Dentistry, University of Leipzig, 04103 Leipzig, Germany; christian.hirsch@medizin.uni-leipzig.de

**Keywords:** oral health, dental caries, obesity, physical activity, periodontal health status

## Abstract

This study aimed to investigate associations between psychosocial factors, obesity, and oral health in a study population of 10- to 18-year-old adolescents who participated in the LIFE Child study. Psychosocial information (socioeconomic status (SES) based on parents’ education, occupation and household income, Strengths and Difficulties Questionnaire (SDQ), health-related quality of life) and physical activity behavior were obtained. Nutritional status was classified based on age- and sex-adjusted body mass index into underweight, overweight, normal weight and obese. Clinical dental examinations were performed and scored with respect to caries experience (CE), oral hygiene (OH), and periodontal status (periodontal health score: PERIO-S). Age-adjusted regression analysis under the assumption of a double Poisson distribution was performed with and without adjusting for SES (α = 5%). A total of 1158 study participants (590 girls, 568 boys; mean age 13.2 ± 2.3 years) were included (17.2% were classified as obese). CE was 20% higher for moderate and 60% higher for low SES compared to high SES (*p* < 0.05). PERIO-S was 10% higher for moderate and 30% higher for low compared to high SES (*p* < 0.05). Poor OH was associated with higher CE (Ratio R = 2.3, *p* < 0.0001) and PERIO-S (R = 3.1, *p* < 0.0001). Physical activity in a sports club was associated with lower CE-S and PERIO-S (R = 0.85, *p* < 0.001). Obesity was associated with increased CE (R = 1.3, *p* < 0.001) compared to normal weight. For low but not high SES, more reported difficulties were associated with higher CE. In conclusion, low SES, poor OH, and obesity are associated with unfavorable oral health conditions, whereas physical activity and high SES are potentially protective.

## 1. Introduction

Dental caries of permanent teeth is among the most common ailments worldwide [1]. However, when it comes to “oral health”, periodontal diseases must also be considered, which rank just a few places behind dental caries in the Global Burden of Diseases Study [1]. Dental caries, gingivitis [2], and periodontitis also occur in children and adolescents, who represent a population of particular importance, especially with regard to preventive measures.

Accordingly, research aims to identify risk factors characterizing unfavorable oral health conditions in adolescents, in order to develop better prevention strategies and deploy them in a targeted way [3]. Lifestyle habits have been shown to play an essential role in caries [3] and periodontal disease [4] development in adolescents. These habits are related to psychosocial determinants and socioeconomic status as prime factors [3]. In the highly industrialized country of Germany, a decline in caries experience was seen in recent decades across all age groups, but especially in adolescents [5]. Notably, caries is not homogeneously distributed, even in populations with the same sociodemographic status [6]. Identifying the reasons for this and, thus, further factors influencing dental caries and periodontal inflammation is essential, because they would help to identify at-risk groups and better target preventive measures. Unfavorable oral hygiene behavior by adolescents, resulting in biofilm accumulation, has been shown to be causative of both dental caries [7] and periodontal inflammation [8]. Furthermore, a higher prevalence of periodontal diseases, especially gingival inflammation, was revealed in obese compared to normal-weight children and adolescents [9,10]. Similar results have considered the association between physical activity and better oral health, especially with respect to periodontal conditions, in children [11] as well as adults [12,13].

Across all age groups, obesity and overweight are worldwide epidemics [14]. Dental caries, periodontal diseases, and obesity share similar characteristics: all of them are of global importance, with behavioral habits (e.g., sugar consumption, unfavorable health behavior) and psychosocial conditions (e.g., socioeconomic status, parental support) reported to be risk factors [3,9,15]. Thus, all three diseases exist within the same multifactorial context, and interactions between them can be expected. Both dental caries and obesity are preventable, non-communicable diseases [3]. Previous studies have considered psychosocial determinants, nutritional status, and caries experience among children and adolescents [3,16,17,18,19], finding associations between caries and obesity. However, the results are quite inconsistent with respect to the social determinants that are shared risk factors for both diseases. Subsequently, more research is necessary to understand the interactions between psychosocial factors, obesity, and oral health conditions. Furthermore, dental caries and periodontal diseases in adolescents seem to influence each other, as recently reported by Carmo et al., who also found further associations with obesity and some other factors [20]. There is a lack of studies considering obesity and oral health status, comprising periodontal signs of inflammation as well as caries, in the same group of adolescents.

It is hypothesized that caries experience and periodontal health condition are associated with (1) psychosocial determinants, (2) behavioral factors (e.g., oral hygiene, physical activity), and (3) the general health condition of obesity after adjusting for socioeconomic status. Thus, the present study aims to examine the distribution of caries and early signs of periodontal inflammation, as well as their associations with nutritional status, psychosocial factors and demographic characteristics, in a cohort of adolescents participating in the LIFE Child study, a population-based cohort study in central Germany [21,22]. 

## 2. Materials and Methods

Adolescent LIFE Child study participants between 2011 and 2015 were included in this investigation. This population-based cohort study (clinical trial number NCT02550236) has been previously described in detail [21,22]. The study followed the Declaration of Helsinki and was approved by the Ethics Committee of the University of Leipzig (Reg. No. 264-10-19042010). Recruitment of children and adolescents took place in schools, medical institutions (e.g., hospitals, gynecologists, public health centers) and via social media [21]. Clinical dental examiners were trained at the beginning of the study and their ratings were regularly calibrated. Four clinical examiners were involved in the study. The mean interclass correlation (ICC) for the ICDAS examination at smooth and occlusal surfaces was 0.9 and 0.8, respectively. ICCs for the periodontal examination were median >0.7. Overall, all ICC values suggested high to very high reproducibility.

### 2.1. Nutritional Status Assessment

For each study participant, weight and height were recorded. Body mass index (BMI) was calculated. To assess nutritional status, BMI was transformed into age- and sex-adjusted standard deviation scores and subsequently classified into “underweight” (BMI-SDS < −1.28), “normal weight” (−1.28 ≤ BMI-SDS < 1.28), “overweight” (1.28 ≤ BMI-SDS < 1.88), and “obese” (BMI-SDS ≥ 1.88), according to the guidelines of the Working Group on Childhood and Adolescents of the German Obesity Association and the German Society of Pediatrics and Adolescent Medicine [23]. The BMI-SDS represents the individual BMI’s standard deviation from the expected median BMI adjusted for age and gender.

### 2.2. Oral Hygiene Assessment

Oral hygiene (OH) was measured at the subject level according to the method of Greene and Vermillion, who classified OH according to plaque and calculus accumulation [24,25] as follows:

Good—no plaque or calculus;

Fair—localized presence of plaque and/or calculus;

Poor—generalized presence of plaque and/or calculus.

### 2.3. Dental Caries Assessment

The DMF index at the tooth level without specification on the surface level was used to investigate dental caries experience. A tooth was classified as a DMF tooth if one of its surfaces was carious or filled (D component: tooth decay because of caries; M: missing because of carious destruction; F: filled surface). As recommended by the WHO, caries diagnosis was based exclusively on visual diagnostics [26]. The components DMF-D (only carious teeth), DMF-M (only missing teeth), and DMF-F (only filled teeth) were also analyzed for a more differentiated analysis.

Additionally, for the six index teeth—16, 11, 26, 36, 31, and 46—all surfaces (occlusal, oral, buccal, mesial, distal) were visually inspected using the ICDAS (“International Caries Detection and Assessment System”, ICDAS 2005) codes [27]. Fundamentally, the choice of index teeth was inspired by the Ramfjord teeth [28], but considering the age of the sample, associated with mixed dentition with the premolars still missing or erupting at examination time, the first molars were considered instead of premolars. The considered sites were diagnosed as 0 = sound; 1 = first visual change in enamel; 2 = distinct visual change in enamel; 3 = localized enamel breakdown; 4 = non-cavitated surface with dentin shadow; 5 = distinct cavity with visible dentin; 6 = extensive cavity with visible dentin. If one of the index teeth was missing, the neighboring tooth was included in the assessment. In addition to caries assessment, existing fillings for the index teeth were recorded. Based on these examination results, “caries experience” was scored (CE-S) by summing up the single tooth scores, with a maximum CE-S of 12. A tooth was given a score of 1 if one surface had a carious lesion according to ICDAS Codes 1 to 4. A score of 2 was given if: (i) at least one surface exhibited a carious lesion according to ICDAS 5 or 6, or (ii) more than one surface had an ICDAS 1 to 4 or (iii) a filling.

### 2.4. Periodontal Assessment

For the six index teeth mentioned above, the Community Periodontal Index of Treatment Needs (CPITN) [29] was classified: CPI 0—healthy situation; CPI 1—bleeding on probing; CPI 2—calculus or plaque retention factor; CPI 3—shallow pocket (4–5 mm); CPI 4—deep pocket 6 mm or more. The following scoring rubric (PERIO-S) was applied for statistical evaluation:

Score of 1: tooth classified as CPI 1 or 2.

Score of 2: tooth classified as CPI 3 or 4.

Scores for the six considered teeth (16, 11, 26, 36, 31, 46) were summed up into the variable “PERIO-S”; thus, a maximum score of 12 could be reached.

### 2.5. Oral Health Score

The oral health score is defined by the authors as the sum score of CE-S and PERIO-S. Thus, it can reach a maximum score of 24 and combines the findings of the periodontal and cariological examinations. This score was included to combine the cariological and periodontal findings and was used for descriptive purposes.

### 2.6. Questionnaire Survey

All participants answered questionnaires about their socioeconomic status (SES), physical activity, and psychosocial factors using the self-report version of the “Strengths and Difficulties Questionnaire” (SDQ) and “KIDSCREEN-27: Quality of life questionnaire for children and adolescents” [30]. The survey was computer-based and took place in a separate room without any time limitation.

The SDQ is a screening instrument measuring behavioral strengths and difficulties [31]. It consists of five scales (emotional problems, conduct problems, hyperactivity/inattention problems, peer relationship problems, and prosocial behavior), each comprising five items. Responses are given on a 3-point Likert scale, resulting in scores ranging from 0 to 10 for each scale. Higher scores indicate more behavioral strengths (prosocial behavior) or difficulties (all other scales). The total difficulties score is the sum of the four problem scores.

The KIDSCREEN-27 is a questionnaire assessing the health-related quality of life of children and adolescents [30]. It consists of five scales. Here, only the Autonomy and Parent Relation scale was analyzed. This scale comprises seven items. Responses are given on a 5-point Likert scale (1—never; 2—seldom; 3—quite often; 4—very often; 5—always). Final scores range from 7 (all items answered with 1) to 35 (all items answered with 5), with higher scores indicating more satisfaction with one’s relationship to one’s parent(s). The score is transformed to age-specific T-values (Mean = 50, sd = 10), which were used for further analysis.

Physical activity was assessed by asking the children whether and how often they engage a) in organized physical (such as a sports club) and b) non-organized physical activity. Both variables were dichotomized into the two categories “at least once a week” (“yes”) and “less than once a week” (“no”).

SES was represented by a composite score combining information on the parents’ education and occupation as well as the equivalized disposable household income as established by Lampert et al. [32]. SES composite scores ranged from 3 to 21, with higher scores indicating higher SES. These scores can be used to categorize SES as either low, middle, or high, based on cut-off scores established in a representative German sample (low: 3.0 to 8.7; middle: 8.8 to 16.9; high: 17.0 to 21) [32]. In a representative sample, the distribution of low/middle/high SES is expected to be 20%/60%/20% [32].

### 2.7. Statistical Analysis

Data management was conducted with SPSS Statistics (version 24, IBM Corp, Armonk, NY, USA). Statistical analyses were conducted with SPSS Statistics (version 24) and R (version 4.1, R Foundation for Statistical Computing, Vienna, Austria.) [33]. Descriptive statistics are presented as the mean and standard deviation for continuous variables and counts and percentages for discrete variables. The Kruskal–Wallis test and the chi-square test were used for descriptive group comparisons. The three outcomes (PERIO-S, CE-S, oral health score) were modeled as dependent on the following predictors: total difficulties score (SDQ), Autonomy and Parents score (Kidscreen-27), physical activity (organized and non-organized), weight group, OH and SES using generalized additive models for location, shape, and scale [34], assuming a two-parametric double Poisson distribution. Model quality was assessed with different diagnostic plots (residuals against fitted values, a scale-location plot of the square root of the absolute residuals against fitted values, a normal Q-Q plot, and a plot of Cook’s distances against leverage/(1-leverage)). Subsequently, the models were rerun adjusting for SES. All models were adjusted for age and sex. Effects are reported as ratios R = exp(beta), where, e.g., an R of 1.5 corresponds to an increase of 50% if the predictor increases by 1. The significance level was set to alpha = 0.05.

## 3. Results

### 3.1. Study Sample Characteristics

From 2011 to 2015, 1158 adolescents (590 girls and 568 boys) between 10 and 18 years participated in the LIFE Child study and received a dental examination. Table 1 gives an overview of the study sample’s characteristics. Mean age was 13.2 ± 2.3 years overall (boys with 13.1 ± 2.6 years, girls with 13.4 ± 2.4 years, *p* = 0.012; Table 1). No differences were found in BMI-SDS, i.e., nutritional status, between male and female participants (mean BMI-SDS 0.41 ± 1.31 overall, 0.41 ± 1.26 in boys, 0.41 ± 1.35 in girls, *p* = 0.952; Table 1). In total, 751 study subjects (64.9%) were of normal weight, and 199 study subjects were obese (17.2% of participants; Table 1). There was also no difference in socioeconomic status (SES) between girls and boys (*p* = 0.991). Overall, 22.3% of participants were classified as having high SES, 63.1% moderate, and 14.5% low (Table 1).

### 3.2. Caries Experience, Periodontal Health, Oral Hygiene, and Oral Health

The caries experience score (CE-S) was 4.81 ± 3.57 in boys and 4.62 ± 3.80 in girls (*p* = 0.369; Table 1). DMF-T (boys: 0.79 ± 1.58; girls: 1.04 ± 1.97, *p* = 0.021) and F-T (boys: 0.48 ± 1.18; girls: 0.66 ± 1.50, *p* = 0.030) showed boys having lower caries experience than girls. D-T (boys: 0.28 ± 0.84; girls: 0.36 ± 0.95, *p* = 0.154) did not differ between male and female participants (Table 1).

The periodontal health score (PERIO-S) was significantly higher in boys (3.67 ± 2.76) than in girls (3.23 ± 2.62, *p* = 0.006; Table 1). 

Oral hygiene (OH) scores differed significantly between girls and boys, with 8.6% of boys versus 4.2% of girls showing poor OH, and 25.1% of girls versus 16.0% of boys exhibiting good OH (*p* < 0.001). 

Regarding overall oral health, including caries experience as well as periodontal health scores, boys had significantly higher scores (8.45 ± 4.93) compared to girls (7.82 ± 5.00, *p* = 0.030, Table 1).

### 3.3. Caries Experience and Periodontal Condition in Association with Different Confounding Factors (SES; OH; Nutritional Status; Physical Activity, SDQ)

**Figure 1:**
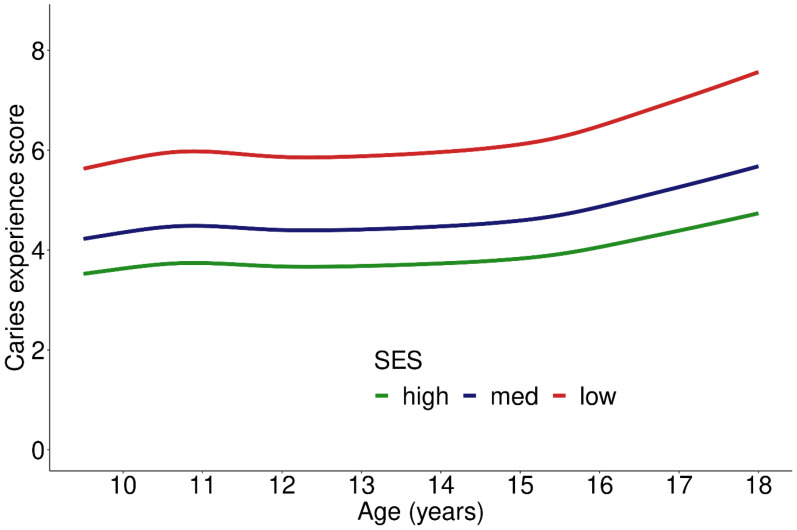
Caries experience score depending on socioeconomic status (SES) across age groups; 905 participants included in the analysis.

CE-S, PERIO-S, and oral health score were positively associated with age, but not statistically significantly so. Nevertheless, all regression analyses were adjusted for age (Table 2). CE-S was 60% higher for low and 20% higher for moderate compared to high SES (R = 1.6, *p* < 0.001 and R = 1.2, *p* = 0.010) (Table 2, Figure 1). PERIO-S revealed 30% and 10% higher scores (R = 1.3, *p* = 0.006; R = 1.1, *p* = 0.042), respectively, when low and moderate SES were compared to high SES (Table 2). Overall oral health showed comparable results, with 40% and 20% higher scores in low and moderate SES compared to high SES, respectively (Table 2). 

**Figure 2:**
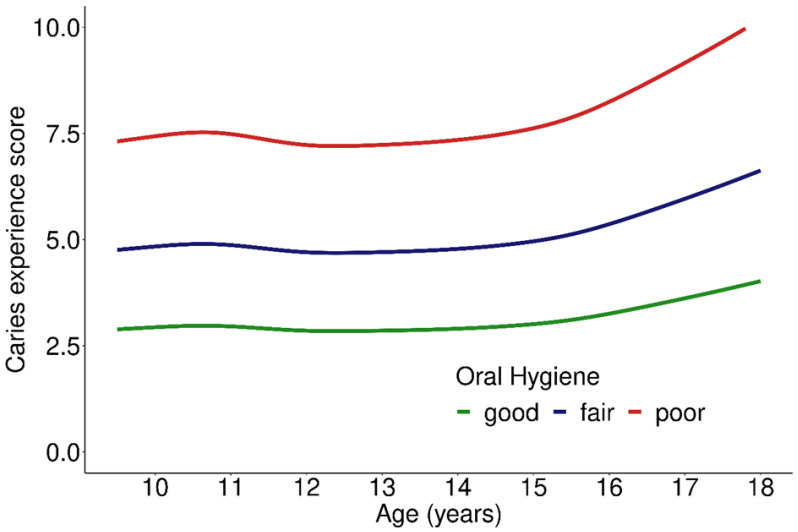
Caries experience score depending on oral hygiene across age groups; 909 participants included in the analysis.

The association between OH and CE-S is illustrated in Figure 2. After adjustment for age and SES, OH exhibited a significant influence, with 60% and 130% higher CE-S, respectively, for fair and poor compared to good OH (*p* < 0.001; Table 2). Furthermore, fair and poor OH were associated with 90% and 210% higher PERIO-S, respectively, than good OH (*p* < 0.001, Table 2).

**Figure 3:**
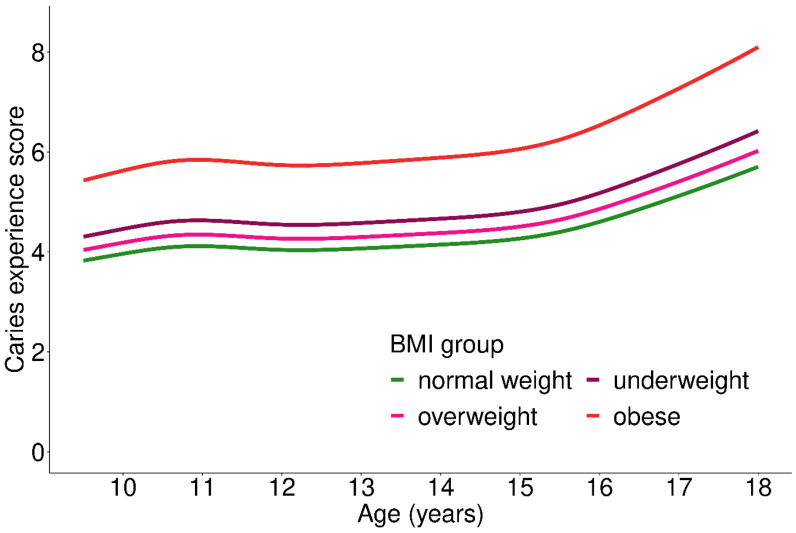
Caries experience score depending on nutritional status (BMI group) across age groups; 1008 participants included in the analysis.

With respect to nutritional status (Figure 3), obese adolescents showed 30% higher CE-S (*p* < 0.001), 20% higher PERIO-S (*p* = 0.028) and 20% higher oral health scores (*p* < 0.001; Table 2) than normal weight adolescents (adjusted for age and SES). Organized physical activity was associated with oral health: physical activity at least once a week in a sports club was associated with 15% lower CE-S and PERIO-S (*p* < 0.001; Table 2). Surprisingly, adjusting for SES only slightly affected the estimate (R = 0.81 vs. R = 0.85). Engaging in sports but not in a club was not significantly associated with either CE-S or PERIO-S (Table 2).

**Figure 4:**
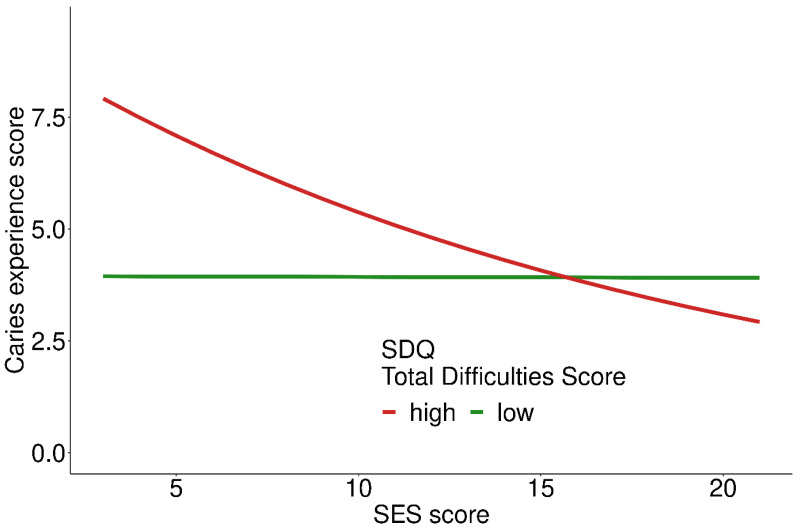
Caries experience score depending on self-reported strengths and difficulties at different SES scores; 905 participants included in the analysis.

For the total difficulties score (TDS), a significant interaction with SES was found (Figure 4). At low SES, high TDS (≥7, more self-reported difficulties) was associated with higher CE-S compared to low TDS (Figure 4). At high SES, the association between TDS and CE-S was absent because CE-S decreased with higher SES (R_SES_) = 0.95/0.93, *p* < 0.001, Table 2). 

**Figure 5:**
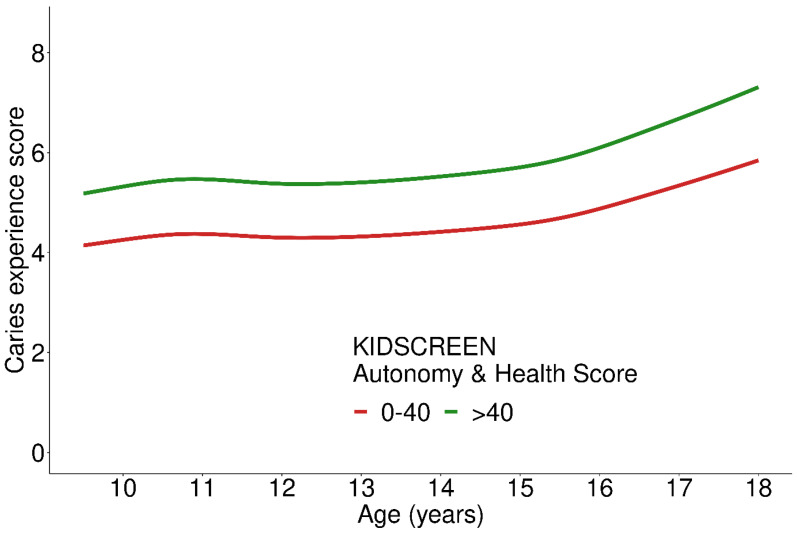
Caries experience score depending on KIDSCREEN Autonomy and Parent Relation score at different ages; 1005 participants included in the analysis.

The Autonomy and Parent score was not associated with either CE-S or PERIO-S after adjusting for SES (Table 2, Figure 5). Subsequently, there was also no association with the overall oral health score.

## 4. Discussion

The results of this study, which aimed at investigating associations between oral health status and obesity as well as psychosocial factors and physical activity in a large study cohort of 10-to-18-year-old German adolescents, reveal a significant association between socioeconomic status (SES) and caries experience score (CE-S) as well as periodontal health score (PERIO-S). Even after adjusting for age, sex and SES, obesity, physical activity, and deficiencies in oral hygiene (OH) were significant determinants of both conditions (CE-S and PERIO-S). An unfavorable total difficulties score in the self-reported strength and difficulties questionnaire (SDQ) was a determinant for CE-S, but only in the case of low SES. In contrast, at low (i.e., better) SDQ scores, no association between caries experience and SES was found.

A study in Portuguese adolescents found female participants to have a higher DMF-T than males [7], which is in line with our findings. The present study observed different results for girls and boys regarding caries experience when analyzing DMF-T and F-T. In contrast, the ICDAS-based CE-S revealed no gender differences. This discrepancy might be attributable to the CE-S threshold of caries detection and inclusion of initial lesions.

Other epidemiological studies have identified gender-based differences in adolescents [35], supporting the present study’s finding that males exhibit less favorable oral health behavior. Several studies revealed a strong relationship between OH and caries experience as well as periodontal/gingival inflammation. Subsequently, associations between OH and CE-S, PERIO-S, and, thus, oral health score were expected. The statistically lower DMF-T and F-T in boys seem inconclusive considering that this group showed less favorable OH. Subsequently, the ICDAS-based CE-S seems more appropriate for analyzing associations between the different influencing factors in the investigated study group. Jablonski-Momeni et al. also concluded that including initial lesions in caries assessment using ICDAS has advantages, especially in populations with low caries prevalence [36].

The main strength of the present study is its consideration of socioeconomic and oral health conditions in adolescents in combination, assessing not only caries experience but also periodontal conditions. In accordance with findings by Kramer et al. [37], the present results reveal that low SES is associated with more caries experience. Another cross-sectional study did not find an association between SES and dental caries in 18-year-old adolescents [19]. A recent meta-analysis found SES in childhood and adulthood to be associated with tooth loss, but concluded that “contextual factors” have to be considered when explaining the effects [38]. This means that personal factors such as genetics and SES cannot fully explain dental health or disease without considering environmental factors (e.g., social environment, food availability) and resulting (health) behaviors as mediators (e.g., hygiene and nutrition, health awareness and health care utilization). Biologically, however, biofilm remains the primary cause of dental caries and periodontal disease (gingivitis/periodontitis). Thus, the pathways from a very unspecific risk factor “unfavorable social environment” [3] to the final endpoint “unfavorable oral microbiome” should be examined. The total difficulties score captures psychosocial problems, which can be considered “intermediary determinants of health” [3]. The present data suggest that in low-SES children, a high self-reported difficulties score was a determinant of caries experience. In contrast, this association was not of relevance in the high-SES group. Similar, potentially protective effects of high SES were found for emotional health among LIFE Child study participants [39].

Besides socioeconomic status, obesity was associated with increased CE-S and PERO-S in the present study. This association is supported in the literature, not only for adolescents [17,40] but also for children [25]. However, the relationship between obesity and unfavorable oral health, including not only dental caries but also periodontal conditions, needs to be further elucidated [3]. 

Considering the observed association between PERIO-S and obesity, the results confirm other studies finding a positive association between parameters of periodontal inflammation (e.g., bleeding on probing, BOP, and probing depth, PD) and obesity. A recent systematic review and meta-analysis showed that the risk of having a PD >4 mm was significantly higher in obese adolescents (OR = 14.15) [41].

Existing data suggest reduced health awareness among obese persons, which may be related to poor oral hygiene behavior [10,42]. However, Vallogini et al. presented conflicting results, finding oral hygiene behavior and periodontal condition to be better in obese adolescents compared to normal-weight patients [43]. From a microbiological point of view, changes in oral microbiota might be associated with obesity, which is believed to induce a low-dose inflammatory burden on the body [44,45]. Immunological responses against invading pathogens and changes in the physiological microbiome are believed to be altered in this process. Consequently, the oral cavity, which represents the first part of the digestive tract, might be affected by microbial changes. On the other hand, changes in the oral microbiome caused by a high frequency of carbohydrate intake [46] might also influence the gut microbiome.

Physical activity is another important factor that has been considered due to its beneficial effects on physical as well as mental health status [47]. The present results reveal a protective effect of physical activity in a sports club on CE-S and PERIO-S. For periodontal health, this association has also been found in children [11] as well as adults [12,13]. Considering dental caries, Stangvaltaite-Mouhat et al. [3] report similar results, with participating in a sports program negatively associated with untreated dental caries [3]. In contrast, Petrini et al. did not find an association between playing organized sports and decayed teeth [11]. Interestingly, the association between physical activity and favorable oral health was not altered when including SES in the model, revealing a general, SES-independent beneficial effect.

Alongside its strengths, the present study followed a cross-sectional design, limiting the findings’ significance for deducing causal relationships [48]. Furthermore, partial examination based on index teeth potentially risks underestimating disease. However, especially for mixed dentition and among adolescents ranging in age from 10 to 18 years, considering first molars and incisors is reasonable because these teeth erupt before age 8. Thus, objective and comparable determination without overestimating probing depth or bleeding due to the eruption of teeth and, thus, underestimating ICDAS can be assumed. In the future, longitudinal observational studies could help to confirm the present results and elucidate the importance of the different risk factors and possible causal links between dental caries, periodontal health status, and obesity. Furthermore, nutrition behavior was not available in this study. Thus, this intermediate determinant could not be considered, limiting conclusions about an essential aspect of health behavior. Subsequently, a standardized questionnaire should be included in a follow-up or any kind of further study. 

The present study revealed a clear association between obesity and oral health condition. Socioeconomic status was considered and found to be determinant as well. The results indicate a possible relationship between dental caries, periodontal health status and obesity, all of which are medical conditions of global importance. They share many similarities in terms of risk factors and are closely related to the digestive tract. Psychosocial risk factors represent another area of common ground, and may define a population of adolescents in particular need of care to prevent dental caries, periodontal inflammation, and obesity. Early identification of high-risk populations in adolescence, or even better, childhood, would allow for targeting preventive measures, resulting in less need for secondary interventions at younger ages and fewer general health problems along the further life course.

## Figures and Tables

**Table 1:** Individual characteristics of the full study cohort (1158 adolescents): 590 girls and 568 boys.

Characteristics	Complete Study Cohort (*n* = 1158)	Boys (*n* = 568)	Girls (*n* = 590)	*p* (Boys vs. Girls)
**Age**, mean (SD) (years)	13.20 (2.30)	13.10 (2.60)	13.40 (2.37)	0.012
**BMI-SDS,** mean (SD)	0.41 (1.31)	0.41 (1.26)	0.41 (1.35)	0.952
**Nutritional status**				0.844
normal weight, *n* (%)	751 (64.90)	374 (65.80)	377 (63.90)	
obese, *n* (%)	199 (17.20)	96 (16.90)	103 (17.50)	
overweight, *n* (%)	108 (9.33)	52 (9.15)	56 (9.49)	
underweight, *n* (%)	93 (8.03)	44 (7.75)	49 (8.31)	
missing, *n* (%)	7 (0.60)	2 (0.35)	5 (0.85)	
**SES composite score,** mean (SD)	12.50 (3.41)	12.50 (3.39)	12.60 (3.44)	0.803
**SES category**				0.991
high, *n* (%)	240 (22.30%)	118 (22.50%)	122 (22.20%)	
moderate, *n* (%)	678 (63.10%)	330 (63.00%)	348 (63.30%)	
low, *n* (%)	156 (14.50%)	76 (14.50%)	80 (14.50%)	
**Caries Experience Score,** mean (SD)	4.71 (3.69)	4.81 (3.57)	4.62 (3.80)	0.369
**DMF-T,** mean (SD)	0.92 (1.79)	0.79 (1.58)	1.04 (1.97)	0.021
**D-T,** mean (SD)	0.32 (0.90)	0.28 (0.84)	0.36 (0.95)	0.154
**F-T,** mean (SD)	0.57 (1.35)	0.48 (1.18)	0.66 (1.50)	0.030
**CPITN > 2; *n* (%)**	410 (35.40)	219 (38.50)	191 (32.32)	0.032
**Periodontal Health Score,** mean (SD)	3.44 (2.70)	3.67 (2.76)	3.23 (2.62)	0.006
missing, *n* (%)	6 (1.06%)	6 (1.02%)	12 (1.04%)	
**Oral Health Score,** mean (SD)	8.13 (4.97)	8.45 (4.93)	7.82 (5.00)	0.030
**Oral Hygiene Score,** mean (SD)	1.84 (0.53)	1.92 (0.52)	1.77 (0.52)	<0.001
good, *n* (%)	239 (20.60)	91 (16.00)	148 (25.10)	<0.001
fair, *n* (%)	722 (62.30)	369 (65.00)	353 (59.80)	
poor, *n* (%)	74 (6.39)	49 (8.63)	25 (4.24)	
missing, *n* (%)	146 (12.60)	59 (10.40%)	64 (10.80%)	
**Organized physical activity at least once in a week**				0.094
no, *n* (%)	279 (24.10)	125 (22.00)	154 (26.10)	
yes, *n* (%)	753 (65.00)	372 (65.50)	381 (64.60)	
missing	126 (10.90)	71 (12.50)	55 (9.32)	
**SDQ Score,** mean (SD)	10.2 (5.25)	9.70 (5.17)	10.6 (5.28)	0.004
missing, *n* (%)	146 (12.60%)	74 (13.00%)	72 (12.20%)	
**KIDSCREEN Autonomy and Parent Relation,** mean (SD)	50.9 (9.10)	51.8 (9.13)	50.1 (8.99)	0.002
missing, *n* (%)	11 (1.94%)	12 (2.03%)	23 (1.99%)	

SD = standard deviation; *n* = number of study participants. SES composite scores range from 3 to 21, with higher scores indicating a higher SES category; low: score ranging from 3 to 8.7; middle: score ranging from 8.8. to 16.9; high: score ranging from 17 to 21.

**Table 2:** Results of age- and sex-adjusted regression analysis.

Confounder	Caries Experience Score		Periodontal Health Score		Oral Health Score	
	exp (beta)	*p*	exp (beta)	*p*	exp (beta)	*p*
**Socioeconomic Status (SES)**						
High	(Ref)		(Ref)		(Ref)	
Moderate	1.2	0.010	1.1	0.043	1.2	0.001
Low	1.6	<0.001	1.3	0.007	1.4	<0.001
**Oral Hygiene (OH)** ^#^						
Good	(Ref)		(Ref)		(Ref)	
Fair	1.6	<0.001	1.9	< 0.001	1.7	<0.001
Poor	2.3	<0.001	3.1	< 0.001	2.5	<0.001
**Nutritional Status** ^#^						
Normal Weight	(Ref)		(Ref)		(Ref)	
obese	1.3	<0.001	1.2	0.032	1.2	<0.001
Overweight	1.0	0.894	1.0	0.908	1.0	0.825
Underweight	1.1	0.320	1.0	0.946	1.1	0.444
**Self-reported strengths and difficulties questionnaire (SDQ)** ^#^						
<7	R_SES_ = 1	0.979	R_SES_ = 0.95	0.002	R_SES_ = 0.98	0.063
7–15	R_SES_ = 0.95	<0.001	R_SES_ = 0.99	<0.186	R_SES_ = 0.97	<0.001
>16	R_SES_ = 0.93 *	<0.001	R_SES_ = 0.96	<0.0017	R_SES_ = 0.94	<0.001
**KIDSCREEN Parents & Autonomy** ^#^						
>40	(Ref)		(Ref)		(Ref)	
0–40	1.1	0.123	1.0	0.778	1.1	0.288
**Organized Physical Activity at Least Once in a Week** ^#^						
no	(Ref)		(Ref)		(Ref)	
yes	0.85	0.010	0.84	0.003	0.84	0.003
exp (beta) = R						

^#^ Adjusted for SES; * higher SES associated with lower caries experience score.

## Data Availability

The legal requirements and the given informed consent do not allow public sharing of the dataset. Interested researchers can contact the research data management of the Medical Faculty, University Leipzig: forschungsdaten@medizin.uni-leipzig.de for further information. The dataset ID is PV468.

## References

[1] Vos T., Flaxman A.D., Naghavi M., Lozano R., Michaud C., Ezzati M., Shibuya K., Salomon J.A., Abdalla S., Aboyans V. (2012). Years lived with disability (YLDs) for 1160 sequelae of 289 diseases and injuries 1990–2010: A systematic analysis for the Global Burden of Disease Study 2010. Lancet.

[2] Tengku H T.N.N., Peh W., Shoaib L., Baharuddin N., Vaithilingam R., Saub R. (2021). Oral Diseases and Quality of Life between Obese and Normal Weight Adolescents: A Two-Year Observational Study. Children.

[3] Stangvaltaite-Mouhat L., Furberg A.-S., Drachev S.N., Trovik T.A. (2021). Common social determinants for overweight and obesity, and dental caries among adolescents in Northern Norway: A cross-sectional study from the Tromsø Study Fit Futures cohort. BMC Oral Health.

[4] Petersen P.E., Baehni P.C. (2012). Periodontal health and global public health. Periodontology 2000.

[5] Jordan R.A., Bodechtel C., Hertrampf K., Hoffmann T.J., Kocher T., Nitschke I., Schiffner U., Stark H., Zimmer S., The DMS V Surveillance Investigators’ Group (2014). The Fifth German Oral Health Study (Fünfte Deutsche Mundgesundheitsstudie, DMS V)—rationale, design, and methods. BMC Oral Health.

[6] Nunes A.M.M., Da Silva A.A.M., Alves C.M.C., Hugo F.N., Ribeiro C.C.C. (2014). Factors underlying the polarization of early childhood caries within a high-risk population. BMC Public Health.

[7] Veiga N.J., Pereira C.M., Ferreira P., Correia I. (2015). Prevalence of Dental Caries and Fissure Sealants in a Portuguese Sample of Adolescents. PLoS ONE.

[8] Räisänen I.T., Sorsa T., Tervahartiala T., Raivisto T., Heikkinen A.M. (2020). Low association between bleeding on probing propensity and the salivary aMMP-8 levels in adolescents with gingivitis and stage I periodontitis. J. Periodontal Res..

[9] Martens L., De Smet S., Yusof M.Y.P.M., Rajasekharan S. (2017). Association between overweight/obesity and periodontal disease in children and adolescents: A systematic review and meta-analysis. Eur. Arch. Paediatr. Dent..

[10] Marro F., De Smedt S., Rajasekharan S., Martens L., Bottenberg P., Jacquet W. (2020). Associations between obesity, dental caries, erosive tooth wear and periodontal disease in adolescents: A case–control study. Eur. Arch. Paediatr. Dent..

[11] Petrini M., Costacurta M., Biferi V., Benavoli D., Docimo R., Spoto G. (2018). Correlation between halitosis, oral health status and salivary β-galactosidases and time spent in physical activities in children. Eur. J. Paediatr. Dent..

[12] Al-Zahrani M.S., Borawski E., Bissada N.F. (2005). Increased physical activity reduces prevalence of periodontitis. J. Dent..

[13] Wakai K., Kawamura T., Umemura O., Hara Y., Machida J.-I., Anno T., Ichihara Y., Mizuno Y., Tamakoshi A., Lin Y. (1999). Associations of medical status and physical fitness with periodontal disease. J. Clin. Periodontol..

[14] WHO (2016). Report of the Commission on Ending Childhood Obesity. https://www.paho.org/en/documents/report-commission-ending-childhood-obesity.

[15] Baxevanos K., Menexes G., Lazaridou A., Coolidge T., Topitsoglou V., Kalfas S. (2021). Dental caries and psychosocial factors: Testing a conceptual model in adolescents. Community Dent. Oral Epidemiol..

[16] Gerdin E.W., Angbratt M., Aronsson K., Eriksson E., Johansson I. (2008). Dental caries and body mass index by socio-economic status in Swedish children. Community Dent. Oral Epidemiol..

[17] Tubert-Jeannin S., Pichot H., Rouchon B., Pereira B., Hennequin M. (2018). Common risk indicators for oral diseases and obesity in 12-year-olds: A South Pacific cross sectional study. BMC Public Health.

[18] Marshall T.A., Eichenberger-Gilmore J.M., Broffitt B.A., Warren J., Levy S.M. (2007). Dental caries and childhood obesity: Roles of diet and socioeconomic status. Community Dent. Oral Epidemiol..

[19] Li L.-W., Wong H.M., Gandhi A., McGrath C.P. (2018). Caries-related risk factors of obesity among 18-year-old adolescents in Hong Kong: A cross-sectional study nested in a cohort study. BMC Oral Health.

[20] Carmo C.D.S.D., Ribeiro M., Teixeira J., Alves C., Franco M., França A., Benatti B., Cunha-Cruz J., Ribeiro C. (2018). Added Sugar Consumption and Chronic Oral Disease Burden among Adolescents in Brazil. J. Dent. Res..

[21] Poulain T., Baber R., Vogel M., Pietzner D., Kirsten T., Jurkutat A., Hiemisch A., Hilbert A., Kratzsch J., The LIFE Child Study Team (2017). The LIFE Child study: A population-based perinatal and pediatric cohort in Germany. Eur. J. Epidemiol..

[22] Quante M., Hesse M., Döhnert M., Fuchs M., Hirsch C., Sergeyev E., Casprzig N., Geserick M., Naumann S., Koch C. (2012). The LIFE child study: A life course approach to disease and health. BMC Public Health.

[23] Wabitsch M., Moß A. (2019). Evidence-based (S3) guideline of the Working Group on Childhood and Adolescent Obesity (AGA) of the German Obesity Society (DAG) and the German Society of Pediatrics and Adolescent Medicine (DGKJ). https://www.awmf.org/uploads/tx_szleitlinien/050-002l_S3_Therapie-Praevention-Adipositas-Kinder-Jugendliche_2019-11.pdf.

[24] Greene J.C., Vermillion J.R. (1964). The simplified oral hygiene index. J. Am. Dent. Assoc..

[25] Elger W., Kiess W., Körner A., Schrock A., Vogel M., Hirsch C. (2019). Influence of overweight/obesity, socioeconomic status, and oral hygiene on caries in primary dentition. J. Investig. Clin. Dent..

[26] WHO (2013). Oral health surveys: Basic Methods.

[27] Ismail A.I. (2005). The International Caries Detection and Assessment System (ICDAS II). https://www.sdpt.net/ICDASEnglish.htm.

[28] Ramfjord S.P. (1967). The Periodontal Disease Index (PDI). J. Periodontol..

[29] Ainamo J., Barmes D., Beagrie G., Cutress T., Martin J., Sardo-Infirri J. (1982). Development of the World Health Organization (WHO) community periodontal index of treatment needs (CPITN). Int. Dent. J..

[30] Kidscreen Group Europe (2016). The Kidscreen Questionnaires: Quality of Life Questionnaires for Children and Adolescents: Handbook.

[31] Woerner W., Becker A., Friedrich C., Rothenberger A., Klasen H., Goodman R. (2002). Normierung und Evaluation der deutschen Elternversion des Strengths and Difficulties Questionnaire (SDQ): Ergebnisse einer repräsentativen Felderhebung. Zeitschrift Für Kinder-und Jugendpsychiatrie und Psychotherapie.

[32] Lampert T., Hoebel J., Kuntz B., Müters S., Kroll L.E. (2018). Messung des sozioökonomischen Status und des subjektiven sozialen Status in KiGGS Welle 2. J. Health Monit..

[33] R Core Team (2021). R: A Language and Environment for Statistical Computing.

[34] Rigby R.A., Stasinopoulos D.M. (2005). Generalized additive models for location, scale and shape. J. R. Stat. Soc. Ser. C.

[35] Mattila M.-L., Tolvanen M., Kivelä J., Pienihäkkinen K., Lahti S., Merne-Grafström M. (2016). Oral health-related knowledge, attitudes and habits in relation to perceived oral symptoms among 12-year-old school children. Acta Odontol. Scand..

[36] Jablonski-Momeni A., Winter J., Petrakakis P., Schmidt-Schäfer S. (2013). Caries prevalence (ICDAS) in 12-year-olds from low caries prevalence areas and association with independent variables. Int. J. Paediatr. Dent..

[37] Kramer A.-C.A., Petzold M., Hakeberg M., Östberg A.-L. (2017). Multiple Socioeconomic Factors and Dental Caries in Swedish Children and Adolescents. Caries Res..

[38] Celeste R.K., Darin-Mattsson A., Lennartsson C., Listl S., Peres M.A., Fritzell J. (2022). Social Mobility and Tooth Loss: A Systematic Review and Meta-analysis. J. Dent. Res..

[39] Herrmann J., Vogel M., Pietzner D., Kroll E., Wagner O., Schwarz S., Müller E., Kiess W., Richter M., Poulain T. (2017). Factors associated with the emotional health of children: High family income as a protective factor. Eur. Child Adolesc. Psychiatry.

[40] Li L.-W., Wong H.M., McGrath C.P. (2017). Longitudinal Association between Obesity and Dental Caries in Adolescents. J. Pediatr..

[41] Li L.-W., Wong H.M., Sun L., Wen Y.F., McGrath C.P. (2015). Anthropometric measurements and periodontal diseases in children and adolescents: A systematic review and meta-analysis. Adv. Nutr. Int. Rev. J..

[42] Saito T., Shimazaki Y. (2007). Metabolic disorders related to obesity and periodontal disease. Periodontol. 2000.

[43] Vallogini G., Nobili V., Rongo R., De Rosa S., Magliarditi S., D’Antò V., Galeotti A. (2017). Evaluation of the relationship between obesity, dental caries and periodontal disease in adolescents. Eur. J. Paediatr. Dent..

[44] Borges M.D., França E., Fujimori M., Silva S.M.C., De Marchi P.G., Deluque A.L., Honorio-França A., de Abreu L.C. (2018). Relationship between Proinflammatory Cytokines/Chemokines and Adipokines in Serum of Young Adults with Obesity. Endocr. Metab. Immune Disord.-Drug Targets.

[45] El-Wakkad A., Hassan N.E.-M., Sibaii H., El-Zayat S.R. (2013). Proinflammatory, anti-inflammatory cytokines and adiponkines in students with central obesity. Cytokine.

[46] Tanner A., Kressirer C., Rothmiller S., Johansson I., Chalmers N. (2018). The Caries Microbiome: Implications for Reversing Dysbiosis. Adv. Dent. Res..

[47] Janssen I., LeBlanc A.G. (2010). Systematic review of the health benefits of physical activity and fitness in school-aged children and youth. Int. J. Behav. Nutr. Phys. Act..

[48] Ahrens W., Pigeot I. (2014). Handbook of Epidemiology.

